# uPAR Expression Pattern in Patients with Urothelial Carcinoma of the Bladder – Possible Clinical Implications

**DOI:** 10.1371/journal.pone.0135824

**Published:** 2015-08-20

**Authors:** Line Hammer Dohn, Helle Pappot, Benedikte Richter Iversen, Martin Illemann, Gunilla Høyer-Hansen, Ib Jarle Christensen, Peter Thind, Lisbeth Salling, Hans von der Maase, Ole Didrik Laerum

**Affiliations:** 1 Department of Oncology, Rigshospitalet, Copenhagen, Denmark; 2 The Finsen Laboratory, Rigshospitalet, Copenhagen, Denmark; 3 Department of Pathology, Rigshospitalet, Copenhagen, Denmark; 4 Biotech Research and Innovation Centre (BRIC), University of Copenhagen, Copenhagen, Denmark; 5 Department of Urology, Rigshospitalet, Copenhagen, Denmark; Wayne State University School of Medicine, UNITED STATES

## Abstract

The objective of the present study was to confirm the expression and localisation pattern of the urokinase-type plasminogen activator receptor (uPAR) focusing on its possible clinical relevance in patients with urothelial neoplasia of the bladder. uPAR is a central molecule in tissue remodelling during cancer invasion and metastasis and is an established prognostic marker in various cancer diseases other than bladder cancer. Formalin-fixed and paraffin-embedded tumour-tissue blocks from 186 patients treated with radical cystectomy were analysed. uPAR expression was scored as either negative or positive as well as by the actual score. Separate scores were obtained for cancer cells, macrophages and myofibroblasts at the invasive front and in tumour core. We were able to confirm, in an independent patient cohort, the tissue expression and localisation pattern of uPAR as investigated by Immunohistochemistry as well as a significant association between uPAR positivity and increasing tumour stage and tumour grade. This demonstrates the robustness of our previous and current findings. In addition the association between uPAR positive myofibroblasts and poor survival was reproduced. The highest hazard ratios for survival were seen for uPAR positive myofibroblasts both at the invasive front and in tumour core. Evaluating uPAR expression by the actual score showed a significant association between uPAR positive myofibroblasts in tumour core and an increased risk of cancer specific mortality. Our investigations have generated new and valuable biological information about the cell types being involved in tumour invasion and progression through the plasminogen activation system.

## Introduction

Extracellular proteolysis is crucial during tumour growth, invasion and metastasis, because of its ability to degrade the extracellular matrix allowing the tumour cells to invade the surrounding tissue including the vascular bed. The matrix degradation is catalysed by a pericellular network of interacting proteolytic systems, of which the plasminogen activation system is a central player. The plasminogen activation system converts plasminogen to plasmin, which both directly and indirectly, degrades components of the basement membrane and extracellular matrix [1]. Plasminogen is activated on the cell surface by the urokinase-type plasminogen activator (uPA) bound to its cell surface receptor uPAR. Receptor binding is thus a prerequisite for pericellular plasmin formation, required for tissue remodelling during cancer invasion [1]. uPA and uPAR are up-regulated in various tumours, including urothelial neoplasia of the bladder [2–8]. These components are known prognostic markers, both when measured in tissue and blood from patients with cancer diseases other than bladder cancer [3–5,9–13]. The prognostic value of uPAR has been shown to be dependent on the cell type expressing it in different cancer types [3–5,12]. Our group has recently shown that uPAR, when studied by immunohistochemistry, was highly expressed in tumour tissue from patients treated with radical cystectomy (RC) for urothelial neoplasia of the bladder. uPAR was primarily expressed by myofibroblasts and macrophages in the tumour associated stroma and to a lesser extent by cancer cells. In addition we demonstrated a significant association between uPAR positivity and T stage, as well as a significant association between uPAR positivity in tumour core and short overall survival [2]. Whether high uPAR expression is correlated to poor prognosis in urothelial neoplasia of the bladder needs to be investigated further. The possible prognostic potential of uPAR expression might be useful in selection of patients with aggressive, highly invasive tumours that could benefit from additional chemotherapy or more intensive follow-up after cystectomy.

Given the potential clinical relevance of uPAR expression in bladder cancer tissue, the purpose of the present study was to confirm our previous findings in an independent patient cohort.

## Materials and Methods

### 2.1. Patient material

Retrospectively collected, routine formalin-fixed and paraffin-embedded (FFPE) tumour tissue blocks from 186 consecutive patients treated with RC and bilateral pelvic lymphadenectomy during the period 2000 to 2005 at a single academic centre (Department of Urology, Rigshospitalet, Copenhagen, Denmark) were analysed. Eligible for inclusion were patients (age 18+ years) with histopathological proven urothelial neoplasia of the bladder. Indications for RC were high-risk non-muscle invasive or muscle invasive disease without signs of lymph node or distant metastases. None of the patients received neoadjuvant or adjuvant chemotherapy. Patient characteristics are presented in Table 1. Moreover, in 33 (18%) cases there was no evidence of residual tumour (n = 32) or only carcinoma *in situ* (CIS) (n = 1) in the cystectomy specimen. In these cases, the prior transurethral resection of the bladder specimen was used for immunohistochemistry and evaluation.

**Table 1:** Patient characteristics (n = 186).

Characteristic		N (%)
Median age, years	63 (range 34–74)	
Gender	Male	144 (77)
	Female	42 (23)
Pathological stage	Ta	6 (3)
	T1	36 (19)
	T2	76 (41)
	T3	63 (34)
	T4	5 (3)
Tumour grade	LG	17 (9)
	HG	169 (91)
Lymph node status	N0	139 (75)
	N+	47 (25)
Lymph vascular invasion	No	167 (90)
	Yes	19 (10)
Resection margin	Negative	176 (95)
	Positive	10 (5)
Concomitant CIS	No	150 (81)
	Yes	36 (19)

LG,low grade; HG,high grade; CIS,carcinoma *in situ*

The study was approved by The Committees of Health Research Ethics in the Capital Region of Denmark (H-1-2012-003) and the Danish Data Protection Agency (2007-58-0015). The investigation was carried out according to the Helsinki Declaration II and in accordance with the REMARK guidelines [14].

### 2.2. Pathological evaluation

All cases were histopathologically reclassified by one pathologist (BRI) with expertise in genitourinary pathology. For each patient, the pathologist reviewed all available FFPE tumour samples from the cystectomy and the block representing the deepest invasive site was selected for further evaluation. The criteria by the Union for International Cancer Control were used for pathological staging and the World Health Organisation classification for pathological grading [15,16]. All other histological features were collected from the original histological reports. Pathologic subgroups were defined as organ confined (pT ≤ T2 N0) and non-organ confined (pT ≥ T3 N0 or pTany N+) disease.

### 2.3. Immunohistochemistry and scoring

#### 2.3.1. Antibodies

The polyclonal antibody (pAb) against uPAR has previously been described and validated [17,18]. Monoclonal antibodies (mAb) against pan-CK (clone AE1/AE3), CK7 (clone OV-TL 12/30), CD68 (clone PG-M1), α-smooth muscle actin (α-SMA) (clone 1A4), as well as EnVision horseradish peroxidase Mouse (K4001) and EnVision horseradish peroxidase Rabbit (K4003) secondary antibodies were purchased from Dako (Glostrup, Denmark).

#### 2.3.2. Immunoperoxidase staining

All stainings were performed in the same laboratory by one person. Whole-slide, 3 μm paraffin sections from each of the blocks were mounted on glass slides and deparaffinised with xylene and hydrated through ethanol/water dilutions. Antigen retrieval for uPAR, cytokeratins (CK-pan and CK7 mixed) and CD68 was performed by Protease K (5 μg/μl) in a Proteinase K-buffer (50 mM Tris-HCL, 50 mM EDTA, pH 8.0) at 37°C for 15 minutes. Antigen retrieval for α-SMA was performed at 98°C in TEG-buffer (10 mM Tris, 0.5 mM EGTA, pH 9.0) for 10 min in a T/T Micromed microwave processor (Milestone, Sorisol, Italy). Sections were blocked for endogenous peroxidase activity by incubating in 1% hydrogen peroxide (H_2_O_2_) for 15 minutes and thereafter washed in Tris-buffered saline (TBS-T, 50 mM Tris-HCL, 150 mM NaCl, 0.5% Triton X-100, pH 7.6) and then manually mounted on Shandon racks with immunostaining cover plates (Thermo Shandon, Pittsburgh, PA, USA). The primary antibodies were diluted in Antibody Diluent with Background-Reducing Components (S3022, Dako) and incubated at the following concentrations overnight at 4°C: uPAR 2.8 μg/ml, CK-mix (CK-pan 0.4 μg/ml + CK7 0.5 μg/ml), CD68 0.3 μg/ml and α-SMA 0.4 μg/ml. The primary antibodies were detected with EnVision Rabbit or Mouse reagents for 45 minutes. The sections were then developed with NovaRed (Vector Laboratories, Burlingame, CA) for 9 minutes. Each incubation step was followed by washes in TBS-T. At last, the sections were counterstained using Mayer’s haematoxylin for 30 seconds, and thereafter dehydrated in ethanol and mounted with pertex using a CoverSlipper from Dako.

#### 2.3.3. Scoring system

uPAR expression was determined by semi-quantitative immunohistochemistry.

**Fig 1:**
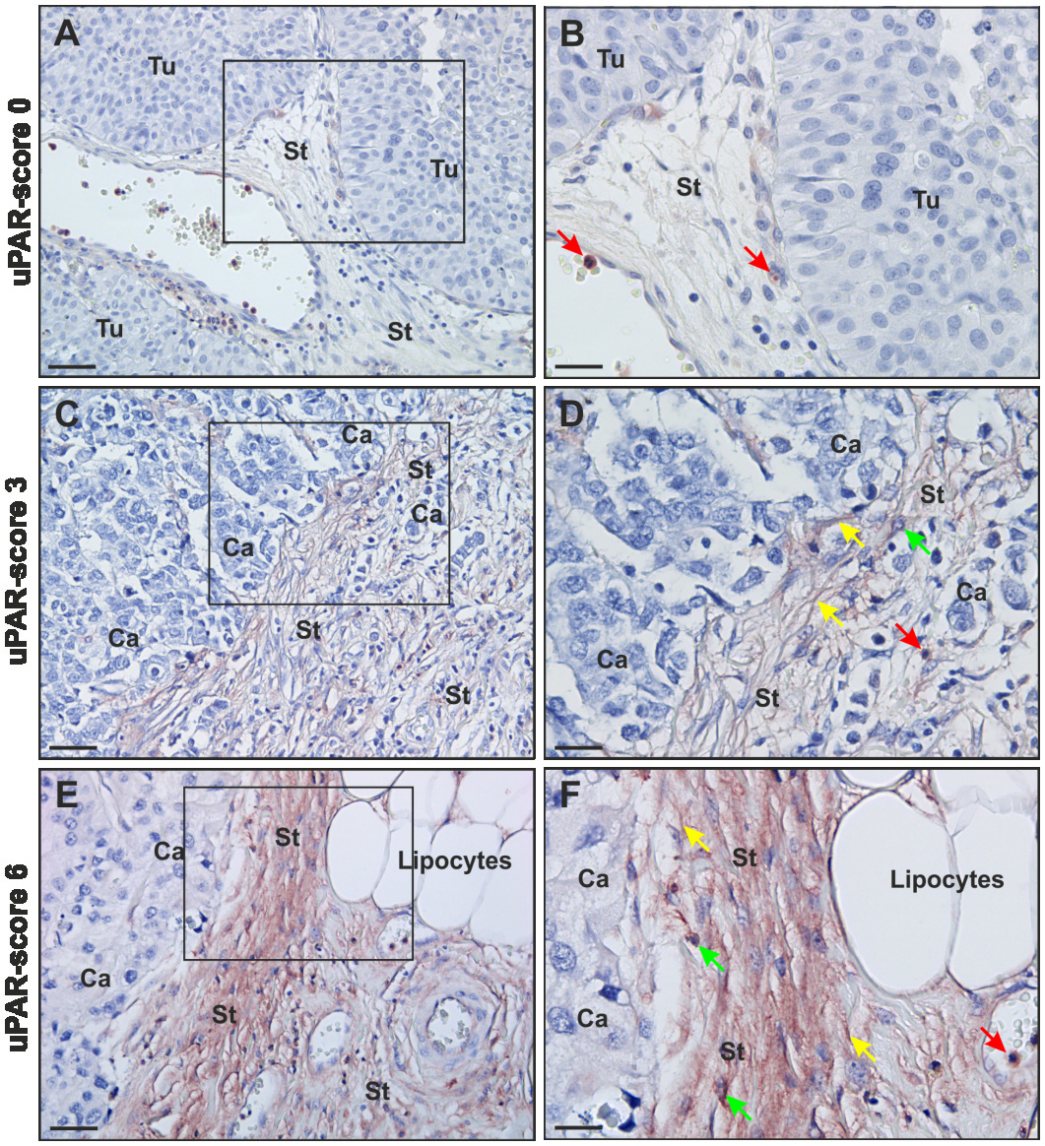
uPAR immunohistochemistry in urothelial neoplasia of the bladder. The figure shows examples of different uPAR scores at the invasive front of the tumour: uPAR score = 0 (no uPAR positive cells detected), uPAR score = 3 (between 5% and 10% positive cells), and uPAR score = 6 (>70% positively stained cells). Tissue sections stained with a pAb against uPAR. uPAR expression was scored semi-quantitatively. The antibody was visualised with NovaRed. uPAR immunoreactivity was scored separately in cancer cells, macrophages and myofibroblasts. The black squares in A, C and E are shown in higher magnification in B, D and F. uPAR immunoreactivity was primarily seen in myofibroblasts (**yellow** arrow in D and F) and macrophages (**green** arrow in D and F) in the surrounding stroma. uPAR positive neutrophils served as internal control (**red** arrow in B, D and F). Tu: tumour, Ca: cancer, St: stroma. Bar in A, C and E ~ 50μm. Bar in B, D and F ~ 25μm.

Neutrophil granulocytes served as positive internal control for uPAR expression [19]. Sections with uPAR negative neutrophils were restained. uPAR immunoreactivity was scored separately in cancer cells, macrophages and α-SMA positive fibroblast-like cells (myofibroblasts) at the invasive front and tumour core, as described previously [3–5]. These cell types were identified in neighbouring sections by immunohistochemical stainings for CKs (cancer cells), CD68 (macrophages), and α-SMA (myofibroblasts). The percentages of uPAR positive cells were scored independently in two locations of the tumour; the deepest invasive front (defined as an up to 0.5 mm wide zone in the tumour periphery at the deepest invasive site) and in the tumour core (everything else but areas of necrosis). The limit of 0.5 mm was based on a pilot study where the zone of invading tumour cells together with an accumulation of macrophages and desmoplasia was within this area (unpublished data). The percentages of uPAR positive cells were grouped into the following categories: 0, no uPAR positive cells detected; 1, less than 1% positively stained cells; 2, between 1% and 5%; 3, between 5% and 10%; 4, between 10% and 30%; 5, between 30% and 70% and 6, >70% positively stained cells. uPAR immunoreactivity was scored blinded by one of the authors (ODL) on coded specimens. Fig 1 shows an example of uPAR score 0, 3, and 6, respectively.

### 2.4. Follow-up

Follow-up after RC was performed according to institutional protocols. In general patients with organ confined disease were seen annually. Patients with non-organ confined disease were seen postoperatively quarterly in year 1 and 2 and semi-annually thereafter.

Follow-up was defined as the interval from cystectomy until death. If no such had occurred data was censored at time of analyses (November 2014). The primary endpoint was overall survival (OS). In addition we explored recurrence free survival (RFS) and cancer specific survival (CSS) [20]. Cancer detection in the ureter or urethra was coded as a second primary cancer and not as a local or distant recurrence. Perioperative mortality (any death within 30 days of surgery) was censored at time of death for CSS analysis. Cause of death was obtained by review of medical records.

### 2.5. Statistics

Descriptive statistics for continuous covariates are presented by the median as well as the minimum and maximum and categorical variables by the frequencies. The associations of dichotomised uPAR scores to clinicopathological covariates were done using the χ^2^-test for independence in the relevant contingency tables. Spearman rank correlation was used as a measure of association between the uPAR scores and tests comparing levels between categories were done using the Wilcoxon rank sum test. Univariate as well as multivariate analysis of time to event data (OS, CSS and RFS) were done using the Cox proportional hazards model. Results are presented by hazard ratios (HR) with 95% confidence intervals (CI). The Cox models have been assessed based on martingale residuals. The uPAR scores have been evaluated by the actual score and dichotomized based on no expression versus a score > 0. In addition, the estimated survival curves based on the Cox regression model for predefined levels of the covariates are presented. The level of significance was set to 5%. All statistical calculations have been done using SAS (v9.3, SAS Institute, Cary, N.C., USA).

## Results

### 3.1. uPAR expression

#### 3.1.1. Urothelial neoplasia and benign urothelium

**Fig 2:**
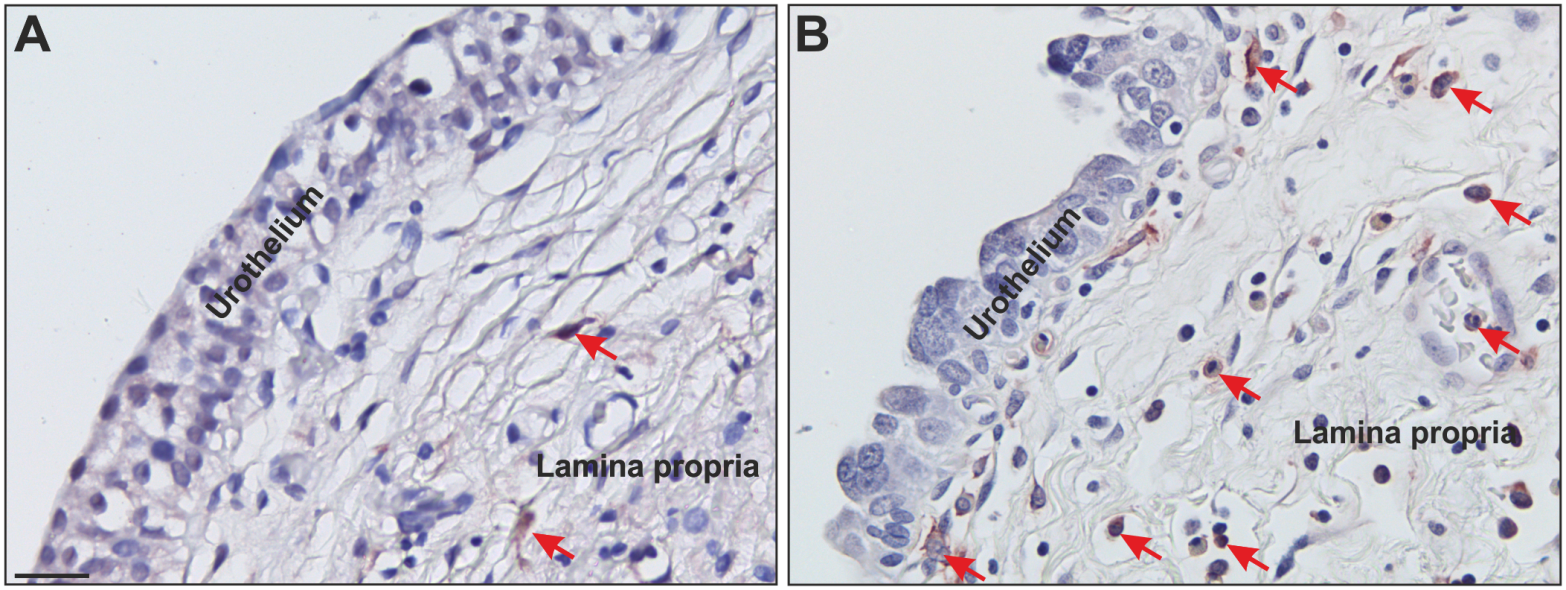
uPAR immunohistochemistry in bladder urothelium with inflammation and CIS, respectively. The figure shows examples of uPAR negative benign-appearing urothelium with inflammation (A) and CIS (B). The tissue section was stained with a pAb against uPAR. The antibody was visualised with NovaRed. The connective tissue (lamina propria) contains leukocytes, of which some few are positive. uPAR positive neutrophils served as internal control (**red** arrow). Bar ~ 25μm.

The invasive front has been analysed in 180 specimens and tumour core in all 186 specimens. As no invasive front is present in non-invasive neoplasias (Ta = 6) these samples were excluded. uPAR immunoreactivity was detected in 173/180 (96%) and 162/186 (87%) of the neoplasias at the invasive front and tumour core, respectively. The cellular source of uPAR was primarily confined to myofibroblasts and macrophages in the surrounding stroma as well as some cancer cells (Table 2 and Fig 1). The adjacent benign-appearing urothelium, as well as cases of concomitant carcinoma *in situ*, were uPAR negative. Fig 2 shows examples of uPAR negative benign-appearing urothelium with inflammation and CIS, respective.

**Table 2:** Univariate analyses. RFS, CSS and OS by uPAR positivity (uPAR score 0 vs. >0)^a^.

			RFS			CSS			OS		
Localisation	Cell type	N (%)	HR	95% CI	p-value	HR	95% CI	p-value	HR	95% CI	p-value
**Invasive front**	All cell types combined										
	No expression	7 (4)	2.66	0.37–19.16	0.33		NA^b^		1.45	0.46–4.56	0.53
	Expression	173 (96)									
	Cancer cells										
	No expression	143 (79)	1.44	0.83–2.51	0.20	1.41	0.78–2.55	0.25	0.95	0.61–1.49	0.83
	Expression	37 (21)									
	Macrophages										
	No expression	9 (5)	2.03	0.50–8.31	0.32		NA^b^		1.42	0.58–3.47	0.45
	Expression	171 (95)									
	Myofibroblasts										
	No expression	13 (7)	2.90	0.71–11.85	0.14	4.80	0.66–34.72	0.12	1.64	0.72–3.74	0.24
	Expression	167 (93)									
**Tumour core**	All cell types combined										
	No expression	24 (13)	1.49	0.64–3.45	0.35	2.06	0-75-5.69	0.16	1.05	0.62–1.78	0.84
	Expression	162 (87)									
	Cancer cells										
	No expression	159 (85)	1.15	0.58–2.25	0.69	1.34	0.68–2.65	0.40	1.03	0.63–1.70	0.90
	Expression	27 (15)									
	Macrophages										
	No expression	31 (17)	1.00	0.52–1.91	0.99	1.52	0.69–3.35	0.30	1.00	0.63–1.58	0.98
	Expression	155 (83)									
	Myofibroblasts										
	No expression	55 (30)	1.67	0.92–3.01	0.09	1.74	0.92–3.28	0.09	1.32	0.89–1.97	0.17
	Expression	131 (70)									

^a^Analyses has been done using the Cox proportional hazards model, and the results presented by the HR with 95% CI.

^b^NA: not accessible because of the limited number of patients with uPAR negative combined with the low event rate.

#### 3.1.2. Association with cell types

**Fig 3:**
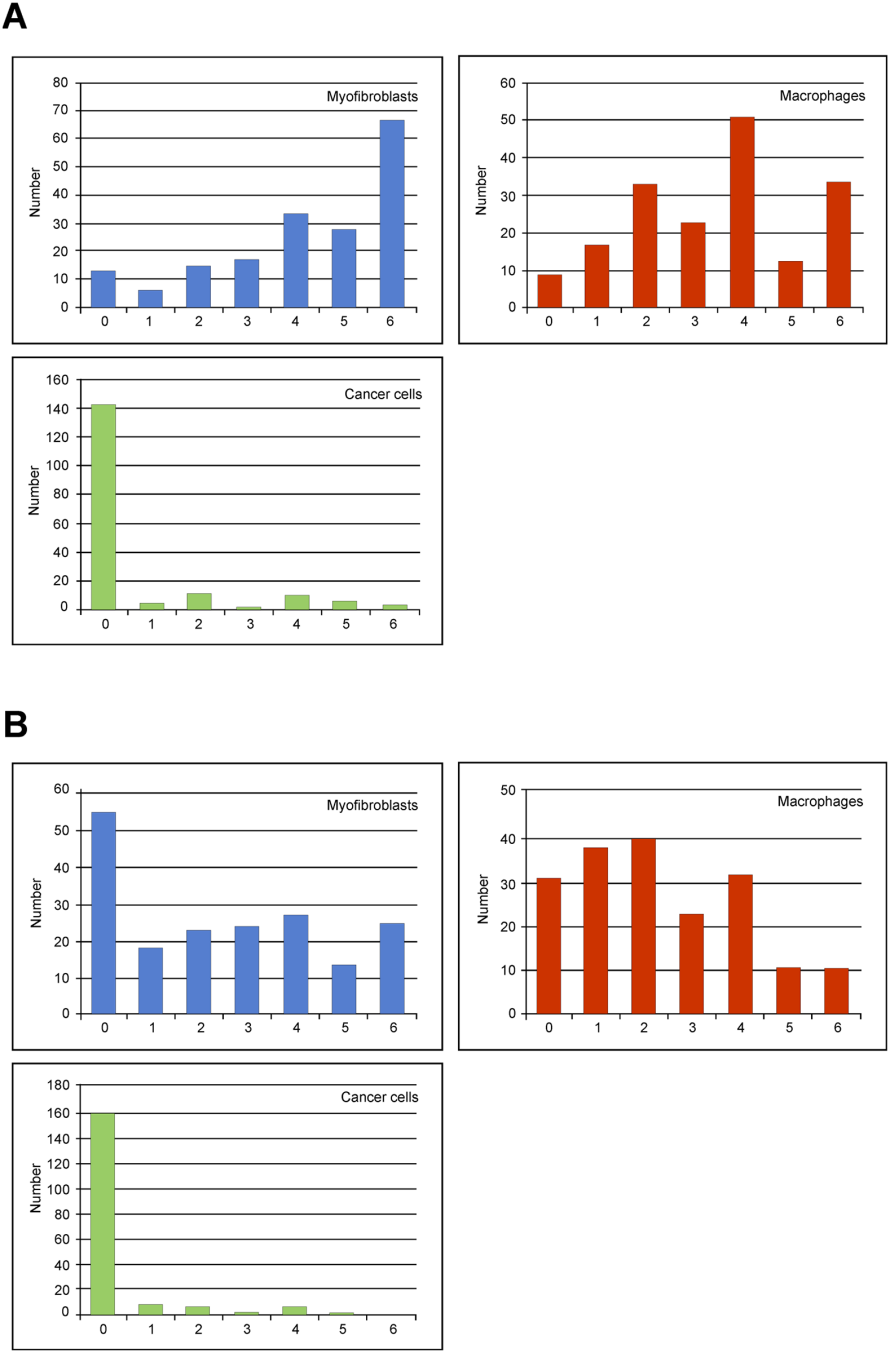
Distribution of the uPAR scores (0–6). Myofibroblasts, macrophages and cancer cells located at the invasive front (A) and tumour core (B), respectively. The x-axis shows the assigned uPAR score and the y-axis the patient number.

The uPAR score (0–6) of the three cell types was assessed and found higher at the invasive front than in tumour core. In both locations the scores for myofibroblasts were the highest (Fig 3A and 3B). Correlation (rs) between uPAR expression at the invasive front and tumour core were 0.45, 0.46 and 0.68 for myofibroblasts, macrophages and cancer cells, respectively.

### 3.2. Association with clinicopathologic features

**Fig 4:**
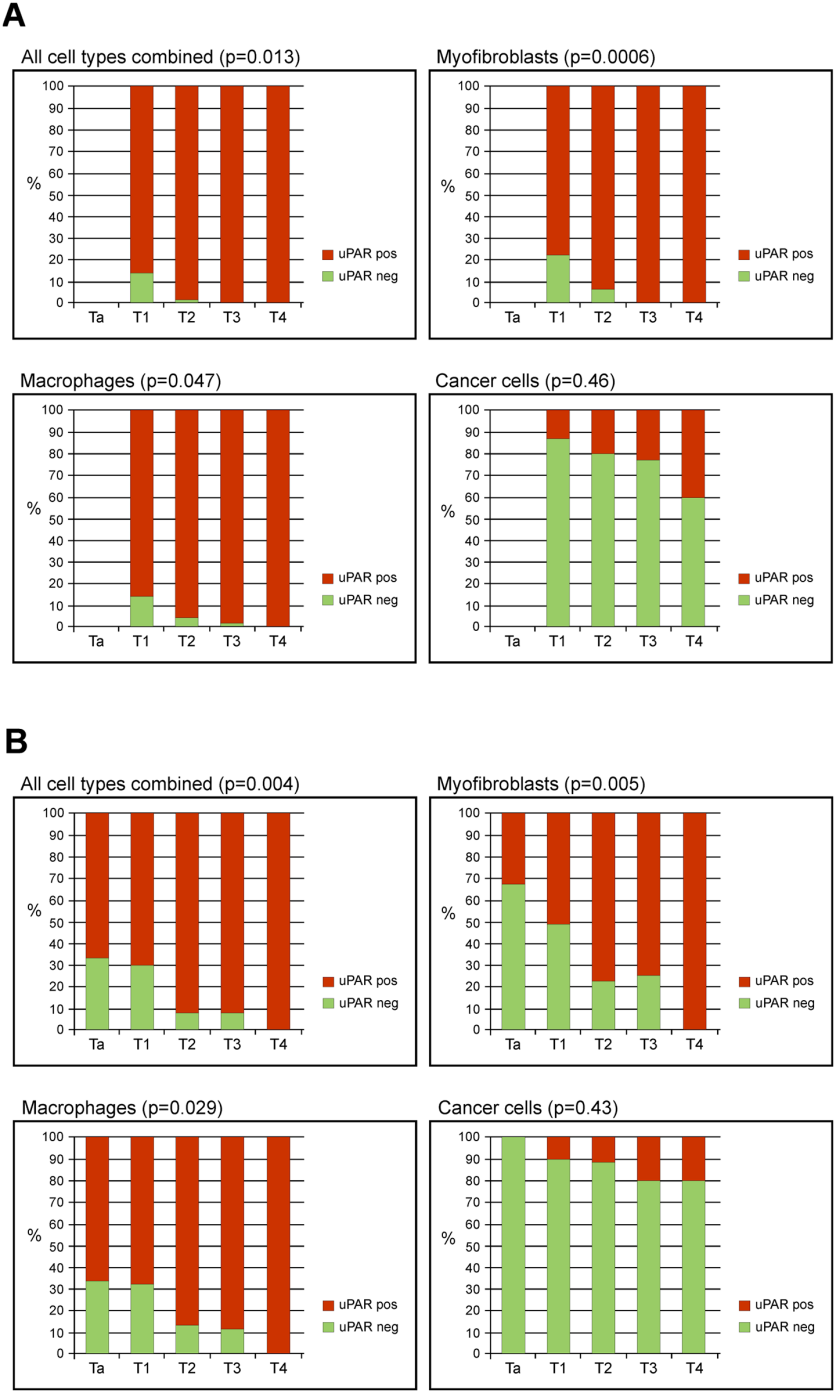
Distribution of uPAR positivity at the invasive front (A) and tumour core (B), respectively. uPAR positivity in both myofibroblasts and macrophages, but not cancer cells, increases significantly with tumour stage. The x-axis shows the tumour stage and the y-axis the percentage of uPAR positive cells. P-values shown are for the χ2-test.

A significant association between uPAR positivity and advanced tumour stage was found for myofibroblasts, and macrophages both at the invasive front and in tumour core (p≤0.015), but not for cancer cells (Fig 4A and 4B). An interaction between cell type and stage could not be demonstrated. In addition, we found a significant association between uPAR positive myofibroblasts and macrophages at the invasive front and higher tumour grade (p = 0.0013, p = 0.0001), uPAR positive myofibroblasts at the invasive front and lymph node metastasis (p = 0.021), uPAR positive myofibroblasts and macrophages in tumour core and lymph vascular invasion (p = 0.014, p = 0.040), as well as between uPAR positive myofibroblasts and macrophages at the invasive front and concomitant CIS (p = 0.026, p = 0.015). No significant association was seen between uPAR expression and gender (p = 0.08 for macrophages at the invasive front and myofibroblasts in tumour core, all other p-values > 0.12) or positive resection margin (all p-values > 0.18). Furthermore, no correlation was found between uPAR expression and age (all p-values > 0.12).

### 3.3. Association with clinical outcome

The 5-year OS, RFS and CSS for the entire cohort were 47.3%, 95% CI: 40.0–54.3; 64.2%, 95% CI: 56.1–71.1; 66.9%, 58.9–73.8, respectively. 65 (35%) patients experienced disease recurrence. 127 patients had died (68%), 57 (31%) of UCB. 3 patients died perioperative. The median follow-up was 11.6 years (range 8.9–14.9) for those patients alive at time of analyses.

**Fig 5:**
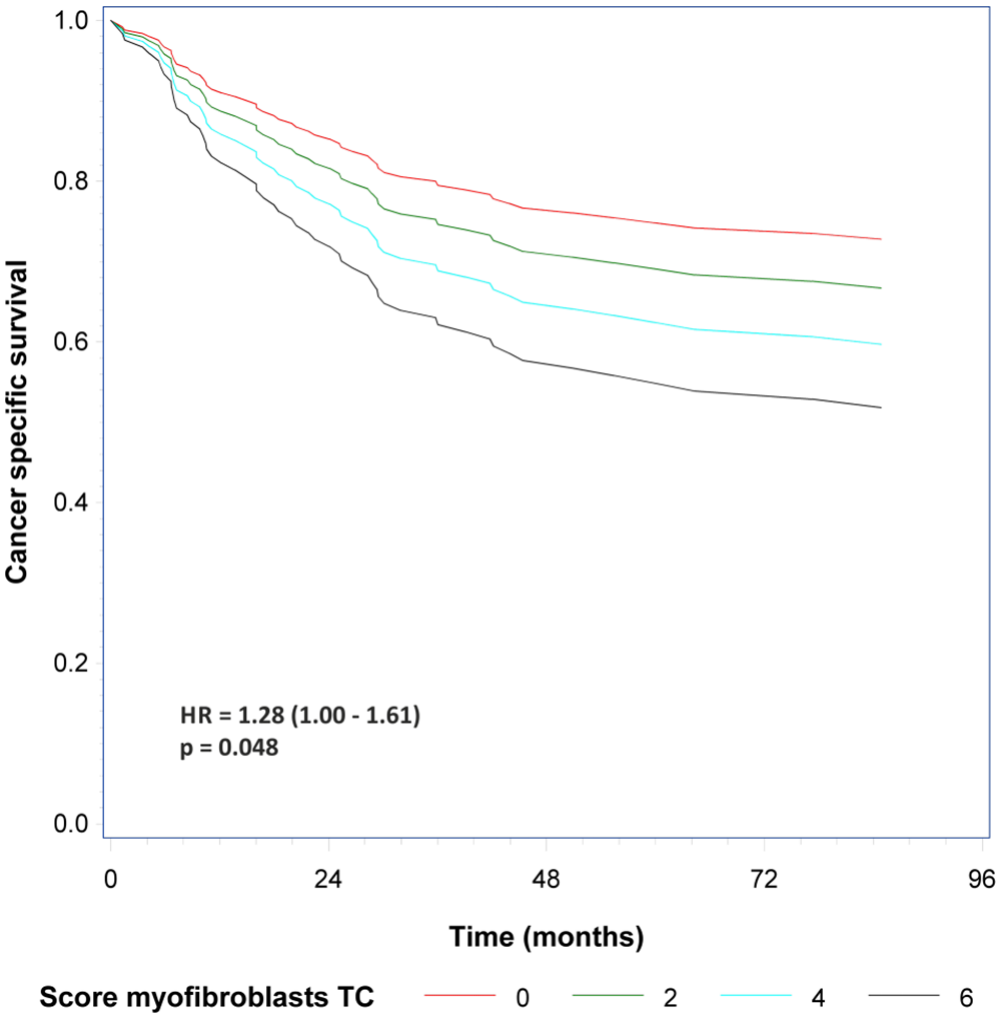
Survival. The figure shows the estimated survival curves based on the Cox regression model for myofibroblasts in tumour core for uPAR score 0 (no uPAR positive cells detected), 2 (between 1% and 5%), 4 (between 10% and 30%) and 6 (>70% positively stained cells). The HR shown is for a 2 unit difference in this score, the p-value is for the score test.

In the univariate analyses no significant association was seen between uPAR positivity and OS, RFS or CSS. The highest HRs for survival was seen for uPAR positive myofibroblasts both at the invasive front and in tumour core (Table 2). To explore this association further the uPAR scores were additionally evaluated by the actual score. This revealed a significant association between uPAR positive myofibroblasts in tumour core and an increased risk of cancer specific mortality (HR = 1.28; 95% CI: 1.00–1.61; p = 0.048) (Table 3, Fig 5). Additionally a trend was seen between high uPAR score in macrophages at the invasive front and longer survival, reaching statistical significance for recurrence free survival (HR = 0.74; 95% CI: 0.56–0.96; p = 0.03). No additional information was uncovered for cancer cells (Table 3). The HRs shown was for a 2 unit difference in this score. No interaction could be shown between tumour stage and uPAR positivity.

**Table 3:** Univariate analyses. RFS, CSS and OS by the actual uPAR score^a^.

		RFS			CSS			OS		
Localisation	Cell type	HR	95% CI	p-value	HR	95% CI	p-value	HR	95% CI	p-value
**Invasive front**	All cell types combined	1.08	0.79–1.51	0.60	1.08	0.76–1.54	0.70	1.06	0.83–1.34	0.66
	Myofibroblasts	1.17	0.85–1.54	0.30	1.10	0.83–1.49	0.49	1.14	0.96–1.35	0.12
	Macrophages	0.74	0.56–0.96	0.03	0.77	0.59–1.04	0.08	0.85	0.69–1.02	0.09
	Cancer cells	1.10	0.83–1.49	0.49	1.10	0.81–1.49	0.56	0.94	0.76–1.21	0.65
**Tumour core**	All cell types combined	1.00	0.79–1.30	0.89	1.12	0.86–1.46	0.40	1.02	0.85–1.21	0.90
	Myofibroblasts	1.19	0.94–1.49	0.14	1.28	1.00–1.61	0.048	1.14	0.96–1.35	0.12
	Macrophages	0.86	0.66–1.14	0.29	0.92	0.69–1.25	0.61	0.88	0.74–1.08	0.25
	Cancer cells	1.02	0.67–1.59	0.90	1.10	0.71–1.72	0.68	0.98	0.72–1.37	0.94

^a^Analyses has been done using the Cox proportional hazards model, and the results presented by the HR with 95% CI. The HR shown is for a 2 unit difference in this score.

Increasing tumour stage (cystectomy specimen), lymph node metastases, lymph vascular invasion and positive resection margin were associated with significantly worse OS (Table 4). No significant association was found between survival and grade, gender or age (Table 4).

**Table 4:** OS. Univariate and multivariate analyses.

		Univariate analysis^a^			Multivariate analysis^a^		
Characteristic		HR	95% CI	P-value	HR	95% CI	P-value
Age per 10 year age difference		1.03	0.81–1.31	0.80			0.80
Gender	Female vs. Male	0.80	0.52–1.24	0.32			0.16
uPAR score of myofibroblasts in tumour core (score 0–6)	HR is for a 2 unit difference in score	1.14	0.97–1.34	0.12			0.62
Tumour stage (cystectomy specimen)	pT ≥ T3 N0 vs. pT ≤ T2 N0	1.66	1.04–2.64	0.033	1.61	1.01–2.56	0.045
	pTany N+ vs. pT ≤ T2 N0	3.28	2.18–4.92	0.0001	2.77	1.80–4.27	0.0001
Tumour grade^b^	HG vs. LG	1.15	0.63–2.09	0.65			0.29
Lymph vascular invasion	Yes vs. No	2.81	1.69–4.67	0.0001	1.87	1.07–3.30	0.0299
Resection margin	Positive vs. Negative	5.41	2.74–10.64	0.0001	2.44	1.15–5.19	0.020

^a^Analyses of overall survival has been done using the Cox proportional hazards model, and the results presented by the HR with 95% CI.

^b^LG,low grade; HGhigh grade.

The significant association between uPAR score and cancer specific survival found in the univariate analysis was not significant in the multivariable analyses (p = 0.41). In the multivariate analyses for the primary endpoint, OS, the statistical significance were retained for the clinical baseline values organ confined disease (HR = 1.61; 95% CI: 1.01–2.56; p = 0.045), non-organ confined disease (HR = 2.77; 95% CI: 1.80–4.27; p = 0.0001), vascular invasion (HR = 1.87; 95% CI: 1.07–3.30; p = 0.030) and positive resection margin (HR = 2.44; 95% CI: 1.15–5.19; p = 0.020).

## Discussion

In the present study we were able to confirm, in an independent cohort of patients with urothelial neoplasia of the bladder, that uPAR as evaluated by Immunohistochemistry is primarily expressed by myofibroblasts and macrophages in the tumour associated stroma. In addition, we were able to confirm the significant association between uPAR positivity in the tumour tissue and increasing tumour stage and tumour grade, which were recently published by our group [2]. This demonstrates the robustness of our previous and current findings. The fact that the cohorts are from two countries and two different time periods emphasises this even further. In addition, we were able to reproduce the association between uPAR positive myofibroblasts and poor survival. Very few tumours showed uPAR negative myofibroblasts and macrophages at the invasive front resulting in possible uncertain estimates of the HRs for all endpoints. As a very high percentage of the uPAR positive myofibroblasts and macrophages at the invasive front and in tumour core were given the score 4 (more than 10% uPAR positive cells) we chose to stratify the scoring system further, compared to our previous study [2], as described in Material and Methods. When evaluated by the actual score a significant association between uPAR positive myofibroblasts in tumour core and poor cancer-specific survival was revealed. In addition we saw a significant association between uPAR positive macrophages at the invasive front and longer recurrence free survival. As highlighted by others [21], our investigations have generated new biological information about the cell types being involved in tumour invasion and progression through the plasminogen activation system.

It is increasingly well accepted that the tumour stroma plays an important role in carcinogenesis. We have in two independent studies shown that in urothelial neoplasia of the bladder uPAR is primarily expressed by myofibroblasts and macrophages and to a lesser extent by cancer cells. In contrast, investigations of its ligand uPA by *in situ* hybridisations have shown that uPA-mRNA is expressed by the malignant urothelial cells themselves [22]. Generally, complex interactions between cancer cells and cells of the neoplastic stroma form a permissive and supportive microenvironment for tumour growth and progression [23]. Data regarding the role of myofibroblasts in bladder cancer is sparse. Our studies stress, however, that these cells in particular seem to play a central role for cancer invasion through the plasminogen activation system in urothelial neoplasia of the bladder, and these results can be directly translated into survival. It is known that tumour associated myofibroblasts secrete a variety of tumour promoting factors including proteases, which contribute to the malignant phenotype [24]. In addition, we found an association between high uPAR score on macrophages at the invasive front and longer survival. It has been hypothesised that tumour associated macrophages can exert dual influence of cancer depending on the activation state, with classically activated (M1) and alternatively activated (M2) cells generally exerting antitumoral and protumoral functions, respectively. Tumour associated macrophages may therefore have fundamental modulating effects on the neoplastic cell population, including tumour cell growth, cell migration, and invasion as well as angiogenesis [25]. We do not have data that can elucidate the subtype of the CD68 positive tumour associated macrophages at the invasive front in our own study.

Urothelial carcinoma of the bladder is an extremely heterogeneous group of malignancies. For patients with high-risk non-muscle invasive and muscle-invasive disease without signs of metastasis, RC with lymphadenectomy is considered the standard treatment. Neoadjuvant chemotherapy is recommended in patients with muscle-invasive node negative disease [26]. Adjuvant chemotherapy is under debate, and is recommended only within clinical trials, but not as a routine therapeutic option [26]. Despite aggressive therapy, patients still suffer from high rates of disease recurrence and shortened survival. Although advances in surgical and oncological treatment, there has been hardly any increase in the survival rate over the past decades. It seems reasonable that a combination of conventional predictors of disease survival and a panel of independent, complementary markers known to contribute to the malignant phenotype might improve significantly the predictive accuracy of standard risk factors such as tumour stage and nodal status and provide improved prognostication for counselling more selectively the use of different treatment approaches *e*.*g*. as to whether a patient should receive adjuvant chemotherapy or to avoid such after cystectomy [27,28]. Despite intensive research of various molecular alterations involved in carcinogenesis of bladder cancer, no molecular markers have been applied successfully in clinical routine practice [29]. The development and validation of biomarkers is a tedious process consisting of a sequence of phases, from discovery to validation and ultimately to assessment of benefit according to the strength of evidence that each provide in favour of the biomarker. The analytical method is the key for obtaining valid and useful results. It is essential that the chosen laboratory method applied is carefully validated according to guideline recommendations [30,31].

uPAR consists of three domains denoted domain I, II and III. uPA bound to uPAR is capable of cleaving neighbouring cell-bound uPAR. Therefore the cleaved uPAR forms reflect the activity of the plasminogen activation system. The cleaved uPAR forms measured in blood have shown to be stronger prognostic biomarkers than the levels of the collective amount of uPAR in several types of cancers [10,13,32–34]; yet, the prognostic value of blood levels of the cleaved uPAR forms in patients with bladder cancer is unknown. In addition to tumour tissue and blood, the association between urinary uPAR and clinical and pathological characteristics, as well as the diagnostic and prognostic potential, have been investigated [35–37]. However, based on current studies, the clinical implications of urinary uPAR seem limited. Our findings indicate that increased uPAR expression in tumour tissue is a marker of invasive and metastatic potential of the tumour at an early stage, and that it is correlated with the clinical outcome of the patients. Based on our studies the prognostic potential of uPAR as investigated by immunohistochemistry in patients with urothelial neoplasia of the bladder does, however, not seem to provide information about clinical outcome independent of standard risk factors such as tumour stage, vascular invasion and resection margin. There might be several reasons for that, but plausible explanations are the semi-quantitative approach for scoring tissue expression of uPAR, as well as the discrepancy between tissue expression of uPAR and biological activity of the plasminogen activation system, due to lack of antibodies that specifically detect the different uPAR forms by Immunohistochemistry. Furthermore, immunohistochemical methods cannot differentiate the separate molecular forms of uPAR. Measurements of biomarkers in blood have in addition obvious clinical advantages compared with tissue samples, such as higher sample homogeneity and minimally invasive nature. Neither blood samples nor fresh frozen tumour tissue are available for the current patient cohort. Based on the present finding the clinical value of the cleaved uPAR forms should be investigated in prospectively collected blood from patients with bladder cancer.

## Conclusion

Our investigations have generated new and valuable biological information about the cell types being involved in tumour invasive and progression through the plasminogen activation system. We were able to confirm our previous published findings of tissue expression and localisation pattern of uPAR, as well as a significant association between uPAR positivity and increasing tumour stage in tumour tissue from patients with urothelial neoplasia of the bladder. In addition we are able to reproduce the association between uPAR positive myofibroblasts in the tumour core and poor survival.

At present, uPAR expression as evaluated by immunohistochemistry cannot be recommended for routine use for prediction of clinical outcome in urothelial bladder cancer. The found biological importance of uPAR in urothelial carcinoma of the bladder may have clinical implications when uPAR forms are measured in body fluids such as blood.
